# Beyond a Diagnosis: A Qualitative Study Exploring Patient and Caregiver Expectations About Emergency Department Visits Amid Uncertainty

**DOI:** 10.1111/acem.70376

**Published:** 2026-07-30

**Authors:** Laura J. Chien, Jennifer Morris, Ann Lawless, Sam Scanlan, Carmel Crock, Maria R. Dahm

**Affiliations:** ^1^ Institute for Communication in Health Care, Australian National University Canberra Australian Capital Territory Australia; ^2^ Independent Patient Safety Advocate Melbourne Victoria Australia; ^3^ Emergency Department Canberra Health Services Canberra Australian Capital Territory Australia; ^4^ Emergency Department Royal Victorian Eye and Ear Hospital East Melbourne Victoria Australia; ^5^ School of Medicine Deakin University Geelong Victoria Australia; ^6^ Centre for Quality and Patient Safety Research, Institute for Health Transformation Deakin University Geelong Victoria Australia

**Keywords:** caregiver, diagnosis, diagnostic uncertainty, emergency medicine, expectations, patient‐centered care, qualitative research

## Abstract

**Objectives:**

Patients presenting to emergency departments (EDs) have been described as expecting a diagnosis or explanation for symptoms, yet diagnostic uncertainty is especially prevalent for those with nonspecific symptoms like headache, dizziness, abdominal pain, and chest pain. Given limited research examining patient and caregiver expectations about diagnosis when uncertainty exists, we aimed to investigate these expectations to inform safe, high quality, patient‐centered diagnostic communication in emergency settings.

**Methods:**

We conducted a qualitative study in two Australian metropolitan EDs using semi‐structured interviews with patients presenting with nonspecific symptoms (*n* = 24) and their caregivers (*n* = 11) about their expectations regarding the visit and diagnosis. We analyzed interview transcripts in NVivo using content and thematic analysis.

**Results:**

Participant expectations about their ED visit and diagnosis were shaped by previous healthcare experiences, understanding of symptom characteristics, familiarity with medical knowledge and uncertainty tolerance. Three themes captured expectations by content: outcomes (understanding, treatment and guidance), processes (diagnostic investigations and being taken seriously), and the health service (timely, integrated care). While most participants reported wanting a diagnosis or explanation, many demonstrated awareness of diagnostic uncertainty and its implications, revealing diverse expectations across a certainty spectrum. Many expected diagnostic investigations, regarding them as integral to determining outcomes but recognized they may not resolve uncertainty. Some regarded EDs as a ‘one‐stop shop’ providing timely diagnostic outcomes. Patients who had previously experienced symptom invalidation expressed strong expectations about being taken seriously.

**Conclusions:**

Patient and caregiver expectations about their ED visit and diagnosis are more nuanced than previously recognized. Findings highlight the importance of clinicians soliciting and responding to hopes and expectations in ways that address the uncertainty inherent in diagnosis. Understanding patient and caregiver hopes and expectations better equips clinicians to communicate in a patient‐centered way about diagnosis and the diagnostic process in EDs, to enhance care even when uncertainty persists.

## 1 Introduction

When patients experience acute symptoms, uncertainty about their meaning and severity can trigger anxiety and fear [1]. To resolve their uncertainty, patients may seek emergency care [2] where they often expect to receive a diagnosis or explanation for symptoms [2, 3, 4, 5, 6]. However, determining the underlying diagnosis can be challenging, particularly when patients present with nonspecific symptoms such as headache, dizziness, abdominal pain and chest pain [7]. These symptoms suggest broad differential diagnoses and create substantial uncertainty [8], reflected in clinical outcomes where over one‐third of ED patients are discharged without a diagnosis explaining their symptoms [9]. While they may receive a symptom‐based diagnostic label [10], patients and caregivers may have lingering and valid concerns about their symptoms [2, 11] and initial reasons for seeking emergency care [3, 12]. Unmet expectations may negatively impact patient experience and satisfaction [13, 14, 15] and pose safety risks including poor adherence to treatment and follow‐up care [14, 16, 17]. Given the importance of expectations in shaping healthcare experiences, investigating patient and caregiver expectations about diagnosis in EDs is crucial to inform safe, high quality, patient‐centered diagnostic communication in emergency care.

Both patients and caregivers (those accompanying patients) hold expectations about healthcare [18], including healthcare processes, outcomes and the broader health system [19, 20, 21]. They are inherently idiosyncratic, influenced by diverse factors [13, 17, 19, 21] and dynamic: they both shape and are shaped by the unfolding clinical encounter [21]. Various frameworks categorize healthcare expectations [13], but most recognize two main components: ‘predicted’ expectations—what patients expect will occur, and ‘ideal’ expectations—what patients want or hope will occur [21, 22].

However, some scholars describe this classification as problematic, arguing that expectations and hopes are distinct constructs: expectations are based on an assessment of what is probable or likely, while hopes reflect what is preferred [18, 23], with hopes linked to greater uncertainty and regarded as more emotionally driven [24]. This distinction matters because patients and caregivers may differentiate between hoped‐for outcomes, which doctors may regard as unrealistic [2, 17], and expected outcomes, and both may influence healthcare interactions and care experience.

Qualitative interview studies investigating expectations about emergency care consistently showed that patients and caregivers expect to receive a diagnosis or explanation for symptoms [2, 3, 4, 5, 6]. Other commonly identified expectations, needs or priorities included receiving diagnostic investigations [2, 4, 5, 6], reassurance [2, 3, 5], symptom relief [2, 3, 5], treatment [2, 5, 6] and planning for next steps [2, 5]. Reviews [25, 26, 27, 28] examining why people seek emergency care have identified common themes related to patient expectations: perceived acuity or urgency and need for reassurance, perceived need for ED/hospital care (diagnostic testing, treatment and medical expertise), and accessibility and convenience.

However, previous studies [2, 3, 4, 5, 6] have not examined distinctions between hopes and expectations [15, 18, 21, 22, 23]—patients may hope for a diagnosis but not expect one—potentially overestimating expectations about diagnosis in EDs. Additionally, some studies did not specify patients' presenting health problem [3, 6] or included diverse presentations (injuries and nonspecific symptoms) [5] making it difficult to determine whether symptom characteristics influenced expectations. Thus, it remains unclear whether patients recognize that nonspecific symptoms are more difficult to diagnose, a notable gap given the prevalence of diagnostic uncertainty in emergency medicine. Furthermore, interview timing varied across studies—during presentation [6], at discharge [2] or up to 30 days post‐discharge [3, 4, 5]—likely affecting responses due to recall bias [29] and expectations evolving over care encounters [13, 29]. These limitations indicate prior research provides only partial understanding of expectations about diagnosis in EDs.

Responding to these limitations, this qualitative study investigated patient and caregiver expectations about diagnosis for patients presenting with selected nonspecific symptoms. By interviewing patients and caregivers before their initial consultation with an ED doctor, we sought to understand expectations situated within the diagnostic uncertainty that often characterizes emergency care.

## 2 Methods

### 2.1 Study Design

This qualitative study is part of a larger multimethod study [30, 31] investigating the impact of communication on diagnostic excellence in emergency care (see protocol [32]). The overarching study combines ethnographic observation, interviews, and discourse analysis of diagnostic interactions to examine communication of diagnostic uncertainty in EDs and received ethical approval from the ethical approval from the ACT Health Human Research Ethics Committee (2022.ETH.00174). No large language model was used to write this manuscript.

### 2.2 The Research Team

Our multidisciplinary team comprises healthcare communication researchers (LJC, MRD), healthcare consumers (JM, AL), and emergency medicine specialists (SS, CC). Our expertise spans complementary perspectives on diagnostic safety: diagnostic communication, lived experience of harm from diagnostic error, and clinical practice.

### 2.3 Study Setting and Participants

We conducted the study in two public metropolitan teaching hospital EDs in Australia: site 1 had approximately 96,000 presentations from July 2023 to June 2024 and site 2 had approximately 51,000 presentations [33]. LJC and MRD recruited a pragmatic convenience sample (first eligible patient to agree to participate) of patients with selected nonspecific symptoms (dizziness, headache, abdominal pain or chest pain) and their caregivers (if present). Prevalent nonspecific symptoms strongly associated with diagnostic error [34] were selected in discussion between two ED Directors (CC, SS) and MRD. After confirming patients' main presenting symptom and triage category with the triage nurse or in the EMR, LJC and MRD alternately approached eligible patients following triage, in the waiting room or ambulance bay depending on arrival mode. Eligible patients were adults triaged as Australasian Triage Scale categories 3 (potentially life threatening), 4 (potentially serious) or 5 (less urgent) [35] with self‐identified sufficient English proficiency (no interpreter services were used). The five‐level Australasian Triage Scale is structurally comparable to the Emergency Severity Index, Canadian Triage and Acuity Scale, and Manchester Triage System [36]. We excluded patients with a history of aggression for safety reasons, people with acute mental health crises or cognitive impairment due to inability to provide informed consent, and pregnant people presenting with abdominal pain due to additional distinct diagnostic considerations. At site 2, patients presenting with chest pain were automatically triage category 2 and so excluded. All participants provided written informed consent.

### 2.4 Data Collection

LJC and MRD collected data at site 1 from August to November 2023 and site 2 from March to April 2024. We anticipated recruiting 24 patients (12 per site), observing and recording patient journeys following triage to admission/discharge [32], limiting recruitment to one patient per day given real‐time ED workflow. Patient and main treating doctor consent were needed for full patient journeys, but patient interviews were retained if conducted before the doctor declined participation. Final sample size was determined by pragmatic and interpretive considerations [37]. Interview location varied by site patient flow; site 1 mostly occurred at the bedside, site 2 in the waiting room, where LJC sat beside participants to maintain conversational privacy. Interviews were conducted in ED settings reflecting naturalistic study design and to avoid disrupting patient care. LJC managed interviews sensitively and flexibly in response to participants' immediate physical and emotional state, checking in with them and pausing as needed for pain, suffering, tiredness and nursing care.

The interview guide was developed by MRD in consultation with JM and members of the Australian National University, Institute for Communication in Health Care, Consumer Reference Group (CRG). CRG members include experienced patient and/or caregiver advocates interested in healthcare communication. LJC piloted the interview guide with the CRG to ensure questions were clearly formulated and interpreted as intended by lay persons [38, 39]. Following pilot testing, we revised questions for clarity, including additional prompts and one new question about previous ED experience. The final guide elicited participants' expectations about their ED visit and diagnosis, and perspectives on communication of diagnostic uncertainty (Supplement S1). This article focuses on expectations about their visit and diagnosis. LJC and MRD collected participant demographic information following consent, assigning each participant a study code comprised of year (23/24), study site (01/02), role (PA‐patient/CA‐caregiver), and identifier (001).

### 2.5 Data Analysis

Interview audio‐recordings were professionally transcribed. LJC reviewed the transcripts for accuracy against original recordings and returned them to participants who had requested to verify transcript accuracy. We used content analysis [40] and iterative thematic analysis [41, 42], supported by NVivo [43]. Using a directed approach [40], LJC developed the initial coding framework based on previous research [2, 3, 5, 44], then deductively and inductively coded five interview transcripts, identifying new codes and refining the coding framework. Patient and caregiver responses were analyzed as individual perspectives rather than relational units. The research team discussed and further refined the framework. LJC coded all interview transcripts. JM and AL independently reviewed LJC's coded data, with discrepancies resolved through discussion. LJC presented data excerpts highlighting hopes versus expectations about diagnosis to the CRG for interpretation. Responding to healthcare consumer i(CRG, JM, AL) input, LJC refined coding to better capture factors shaping expectations (past healthcare experiences, awareness of uncertainty and understanding of ED's role), and observed expectations (including reassurance, being taken seriously, timely access to investigations and specialists, and hopes versus expectations). MRD, JM and AL provided feedback on LJC's preliminary thematic analysis, with healthcare consumers contributing insights into the emotional burden of managing hoped‐for versus likely diagnostic outcomes, need for a path forward and challenges accessing in‐community care, and elements relevant to being taken seriously (perceived thoroughness, explaining reasoning, and reassurance as symptom dismissal).

### 2.6 Trustworthiness

MRD, with extensive experience in qualitative research in healthcare contexts, led the overarching project and oversaw data collection. LJC, a qualitative researcher, conducted all patient and caregiver interviews and led analysis and interpretation of findings with co‐author input, including healthcare consumers and emergency medicine specialists. We used multiple strategies to establish trustworthiness [45] (Table 1). Study procedures were documented in detail [32]. Results reporting follows qualitative research criteria [46] (Supplement S2).

**TABLE 1:** Strategies to establish trustworthiness.

Trustworthiness criteria	Strategies to meet criteria
Credibility	Conducted interviews about diagnostic uncertainty with participants experiencing that phenomenon in a naturalistic setting, strengthening data authenticity. Returned transcripts to participants to verify accuracy. Code and data review by multiple analysts and peer debriefing with CRG members challenged interpretations and assumptions, supporting analytical depth.
Transferability	Pragmatic sampling that captured patients with diverse nonspecific symptoms and caregiver relationships, reflecting ED reality. Information about individual participant characteristics provided in Supplement S3.
Dependability	Maintained detailed audit trails documenting research decisions, data collection procedures and analytical reflections.
Confirmability	Maintained reflective notes throughout data collection and analysis, discussing potential bias and interpretations with team members. Peer debriefing with supervisory panel members.

## 3 Results

We approached 58 patients; 26 patients consented (response rate 45%). Response rates differed by gender: 60% of female patients consented compared to 18% of male patients. The most common reason for declining was feeling unwell. Participating patients (*n* = 24; one withdrew, one excluded before being interviewed because treating doctor declined to participate [32]) were 18–75+ years, predominantly female (*n* = 20; 83%), native English speakers (*n* = 21; 88%) and all had previously presented to an ED. Tables 2 and Supplement S3 provide detailed participant information.

**TABLE 2:** Summary of participant characteristics.

Patients (*n* = 24)	*n* (%)	Caregivers (*n* = 12)	*n* (%)
**Triage category** ^a^			
3	11 (46)		
4	12 (50)		
**Main presenting symptom**		**Relationship to patient**	
Abdominal pain	13 (54)	Spouse/partner	6 (50)
Headache	5 (21)	Adult child	2 (17)
Dizziness	4 (17)	Friend	3 (25)
Chest pain	2 (8)	Paid carer	1 (8)
**Gender**			
Female	20 (83)	Female	8 (67)
Male	3 (13)	Male	4 (33)
Gender diverse	1 (4)	Gender diverse	0 (0)
**Age**			
18–35	8 (33)	18–35	4 (33)
36–65	9 (38)	36–65	5 (42)
66 and over	7 (29)	66 and over	3 (25)
**Language background**			
NES	21 (88)	NES	11 (92)
NNES	3 (13)	NNES	1 (8)
**Ethnicity**			
Oceanian	17 (71)	Oceanian	10 (83)
Indigenous Australian	1 (4)	Indigenous Australian	0 (0)
Other	6 (25)	Other	2 (17)
**Highest education completed**			
(some) high school	6 (25)	(some) high school	1 (8)
Vocational/other	5 (21)	Vocational/other	2 (17)
University	13 (54)	University	9 (75)

Abbreviations: NES, Native English speaker; NNES, Non‐native English speaker.

^a^ One patient triage category is unclear.

LJC conducted 25 semi‐structured interviews with patients (*n* = 24) and caregivers (*n* = 11), comprising 15 individual interviews and 10 interviews with patient‐caregiver pairs. All patient‐caregiver pairs were interviewed together except for one pair, with questions directed to each participant in turn. One caregiver was unavailable for interview (only the patient of that pair was interviewed). All interviews except two took place in the ED before the patient's first consultation with an ED doctor. Five patients reported consulting a GP immediately before or in the days preceding their ED presentation. Interviews ranged from 10 to 33 min duration (average duration: individual interviews: 16 min; patient‐caregiver pairs: 24 min).

**FIGURE 1:**
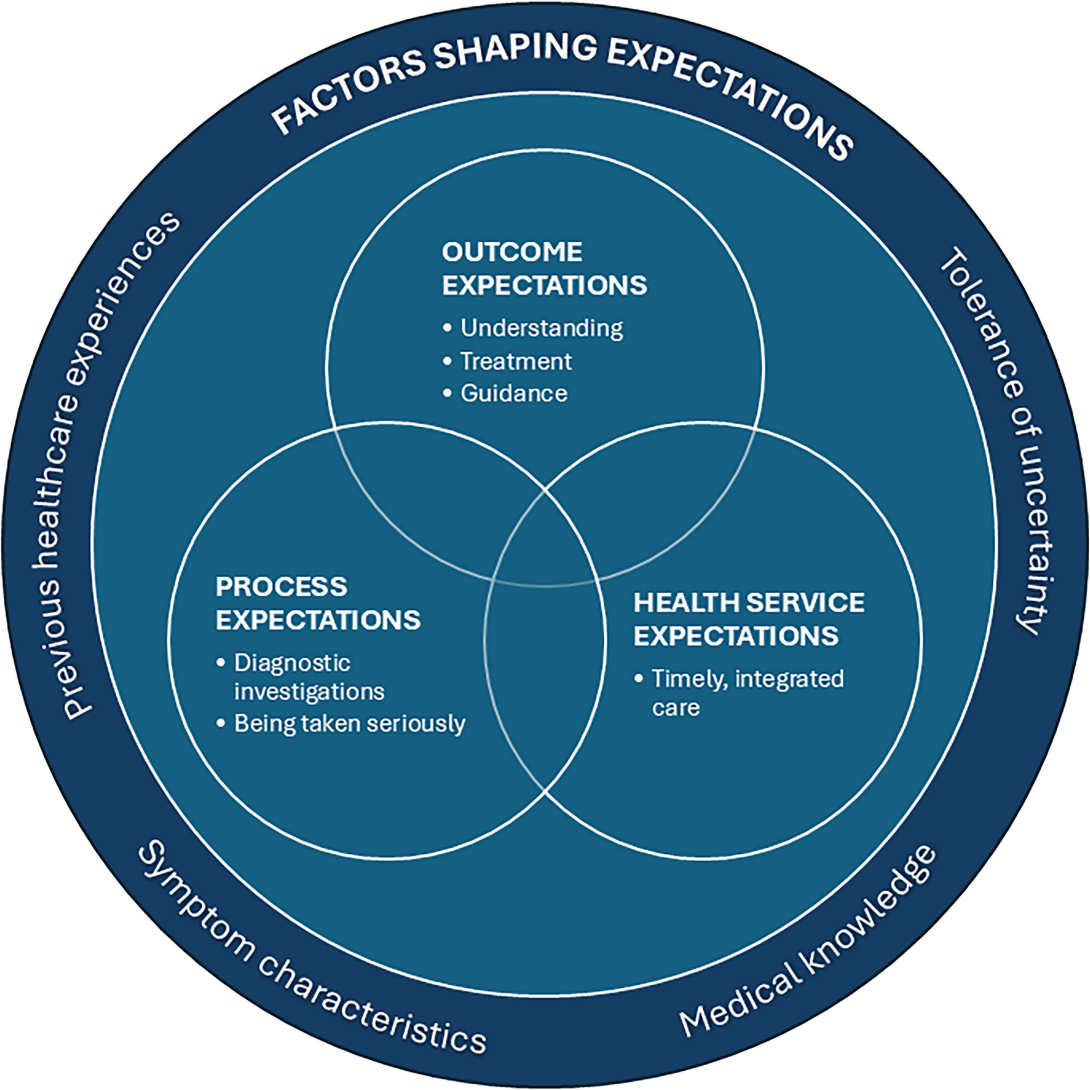
Patient and caregiver expectations about diagnosis in EDs.

Figure 1 provides presents four identified themes. One theme captured factors shaping patient and caregiver expectations, including previous healthcare experiences, understanding of symptom characteristics, medical knowledge, and uncertainty tolerance. Three themes captured patient and caregiver expectations by content [21]: (1) outcomes—the end results of care, including understanding, treatment, and guidance, (2) processes—what doctors do both technically and interpersonally to provide care, including diagnostic investigations and taking patients seriously, and (3) the health service—about the ED itself, including timely, integrated care. Each is detailed below.

### 3.1 Factors Shaping Expectations

Some participants noted factors shaping expectations about their ED visit and diagnosis: prior healthcare experiences, including symptom dismissal due to various biases; understanding of symptom characteristics (e.g., vague, resolved, needing urgent attention); medical knowledge and tolerance of uncertainty (e.g., accepting not having a clear diagnosis).

### 3.2 Outcome Expectations

#### 3.2.1 Understanding

Understanding encompasses knowledge about the patient's health problem through receiving a diagnosis or explanation for symptoms, including what it is not, via ruling out serious conditions.

More than two‐thirds of participants (*n* = 24; 69%) reported wanting a “clear, confirmed diagnosis” (2402PA007) or explanation to “know what is actually happening” (2301CAX02A). However, many participants (*n* = 20; 57%) also recognized a definitive diagnosis or explanation might not be possible, often describing factors contributing to uncertainty. Some expected a working diagnosis: doctors would “only …make suggestions” given resolved symptoms (2402CA004). Others understood they may not receive a diagnosis or explanation: “I'm not really expecting an explanation” given symptom “vagueness” (2402PA008). Some “hop[ed] for a diagnosis” (2301CA008) but did not expect one, acknowledging inherent uncertainty: “Sometimes they just don't know why you're not well” (2301PA008); “I've been around long enough to know that a lot of the time they just don't know, and it takes further tests and research” (2301CA008).

The following patient‐caregiver interaction illustrates the emotional work patients do to manage expectations amid uncertainty:

2301PA011: Why am I hoping for [a diagnosis]? Well, because it's why I'm here because I don't know what this is due to. So I don't know whether it's something serious or it isn't. Whether I can just ignore it and…put up with the pain or whether…something needs to happen…

2301CA011: But you don't have an expectation that would happen, you're just hopeful?

2301PA011: No, I expect it. Yeah, I do expect it.

2301PA011 rationalizes her hope for a diagnosis—whether her symptoms indicate something serious and require action. 2301CA011's question invites 2301PA011 to recognize her need for a diagnosis as legitimate, reframing hope as an expectation.

Almost half of participants (*n* = 15; 43%) wanted doctors to rule out serious diagnoses to provide reassurance about worrying symptoms: “[symptoms] have become more intense…I'm concerned it's not something serious…and wanting that confirmed” (2402CA005).

While some expressed this outcome as hoped‐for: “the best [outcome] would be to find out it's nothing serious” (2301PA002), for others, it reflected expectations about diagnosis in EDs:

I think the ED usually assesses for acute issues, make sure you're okay for the moment, but often gives a preliminary, 'We think it's this, so you should go see [your] GP…' (2301PA007).

Some participants expected doctors to rule out specific feared diagnoses: “I'm worried about it being pancreatitis or something with my liver or gallbladder. I think they'll be able to rule those things out” (2301PA002). Others presented with their own candidate diagnoses, expecting doctors' confirmation: “to find out if it's AFib [atrial fibrillation]” (2301PA012). Some were more pragmatic; 2301PA003 expected “reassurance that…my symptoms are normal cause of [this] virus,” suggesting comfort with uncertainty when suspicions of a nonserious condition are confirmed.

#### 3.2.2 Treatment

More than half of participants (*n* = 19; 54%) expressed expectations about treatment relating to managing symptoms (immediate outcomes) and/or resolving health problems (longer‐term outcomes).

Expectations about treatment for managing symptoms centered on alleviating or controlling symptoms: “for my client to feel better” (2301CA010); “control the pain” (2301PA010); “for 2301PA008 to not be in pain and suffering” (2301CA008). Some participants expected medications specifically: “to stop the spasming” (2402PA008); “because I'm not feeling great” (2301PAX03).

Some connected expectations about treatment to resolve their health problem to diagnostic clarity: “a diagnosis…and…treatment so it goes away” (2301PAX02); “if they could work out that it was AFib…then she could start treatment” (2301CA012). 2402PA009 expected diagnostic clarity as her symptoms were “very uncomfortable and anxiety‐causing,” emphasizing the physical and emotional suffering unexplained symptoms may cause. Others recognized treatment could proceed based on diagnostic hypotheses, with “speculative” treatment response potentially providing diagnostic clarity: “Try this and see if it goes away” (2402CA008); if it is “some kind of infection…I can take antibiotics…and not have to be in pain anymore” (2402PA003).

#### 3.2.3 Guidance

More than one‐third of participants (*n* = 13; 37%) expected guidance about next steps, including to resolve their immediate health problem, further investigate symptoms and address broader concerns. Some viewed diagnostic certainty as a precursor to guidance: “a diagnosis with a plan of attack to rectify it” (2402PA009); “we can rule out these things and therefore it's probably this and this is what we do going forward” (2301CAX02A). More often participants anticipated uncertainty and expected guidance about next steps, viewing EDs as a pathway to ongoing care: “some sort of plan…we don't know what it is. What do I need to do” (2402PA001). 2402PA008 did not “expect an answer today” but “possibly a referral if it's not looking like going away.” 2402PA005 did not think doctors would “know straight away” and anticipated a colonoscopy “to check what this could be.” Caregivers sought guidance about next steps regarding broader concerns: regaining “quality of life” (2301CA008); reassurance that 2402PA004 was “okay to go home” by himself (2402CA004); and “sensible advice” on proceeding with planned fertility treatment (2301CA005).

### 3.3 Process Expectations

#### 3.3.1 Diagnostic Investigations

Almost half of participants (*n* = 16; 46%) expressed expectations about diagnostic investigations, regarding them as integral to determining diagnosis, ruling out serious conditions, and reassurance. 2402PA002 expected a CT scan to “see what's happening with this inflammation in my body”, expecting doctors “to diagnose on that.” 2402CA004 expected reassurance that “nothing serious has happened,” explaining a blood test would detect a slight heart attack, while 2402PA007 expected scanning for “some assurance before…discharging me”.

Some participants recognized that investigations may not resolve diagnostic uncertainty. 2402PA008 did not expect “an answer” if “nothing presents on…standard blood tests.” 2402CA005 expected “progress in making [a diagnosis]” rather than definitive answers:

I guess they go through the process of elimination, and…by the end of the day, certain things have been eliminated, like an infection or because 2402PA005's given a sample…

Participants did not mention history‐taking, physical examination or specialist consultations, though some referred to information gathering generally: “investigate the cause” (2402PA009); “proper checks” (2301CA001).

#### 3.3.2 Being Taken Seriously

Almost one‐third of patients (*n* = 7; 29%) wanted to be taken seriously, particularly those who had previously experienced symptom dismissal due to various biases. They often associated being taken seriously with perceived thoroughness of care. Concerned about a subarachnoid hemorrhage, 2301PA001 stated “making sure that's taken seriously is probably the biggest thing.” 2301PA012 noted her undiagnosed chest pain was often “put down to” her post‐traumatic stress disorder. 2301PA004 wanted to feel “thoroughly attended to” by “somebody who's a professional and understand what could be going on,” rather than dismissed with “Ah well, it's probably anxiety, right?” 2402PA003 approached medical care with skepticism: “We tend to assume that everybody's going to go, ‘Oh well, it's just because you're overweight.’” 2301PA013 hoped doctors “find whatever is wrong with me instead of just saying, ‘Sometimes pain just happens’ and send me on my way.” 2402PA006 found thorough investigation made uncertainty more acceptable: “I'd rather them look into it and then be like, ‘Oh, there's nothing,’ than just kind of guess.”

Two patients whose concerns had not been adequately addressed previously re‐presented to site 1 expecting more thorough investigation. 2301PA009 stated “they've got to do something other than give me a couple pills and say, ‘Go home.’” 2301PAX03 reported her mother hoped “a different doctor has a different opinion, or different knowledge that will look into it a bit deeper.” These patients recognized variability in doctors' diagnostic approaches and expertise, and believed persistence through re‐presenting may lead to improved outcomes.

### 3.4 Health Service Expectations

#### 3.4.1 Timely, Integrated Care

One‐third of participants (*n* = 12; 34%) expressed expectations about EDs delivering timely, integrated care, perceiving them as a ‘one‐stop shop’ providing diagnostic outcomes. 2301PA002 explained it was easier to present to ED “to get everything…dealt with at once” as it was “hard to get in with my GP and coordinate everything.” 2301CAX02A noted in ED “tests which are needed…can be performed. And so there isn't schedule it, schedule it, schedule it and it's 2 weeks later before you can get in everywhere.”

Some believed EDs possessed superior diagnostic capabilities: “they've got all the tests…all the imagery…. They know 2301PA011's history so if not here, I don't know where” (2301CA011). Others valued timely diagnostic outcomes due to concerns symptoms indicated conditions requiring urgent intervention: “if there's anything going on, that it gets dealt with promptly before it escalates” (2402PA005).

GP referrals to ED reinforced ‘one‐stop shop’ perceptions. 2402PA009 reported her GP said, “it's best to do [tests] here rather than…have them separately and wait.” 2402PA002, also referred by her GP, expected a CT scan to confirm her diagnosis: “whether it is a diverticulitis attack or not.”

## 4 Discussion

To our knowledge, this is the first study on patient and caregiver expectations about diagnosis in EDs to interview participants *before* consultation with an ED doctor. This approach captured expectations amid real‐world uncertainty rather than mid‐visit [6] or retrospective accounts [2, 3, 4, 5] potentially shaped by clinical encounters. This study extends understanding by investigating expectations from the perspective of patients and caregivers actively experiencing uncertainty about the cause of nonspecific symptoms, revealing considerable awareness of diagnostic uncertainty. We identified interconnected expectations encompassing ED visit outcomes (understanding, treatment and guidance), processes (diagnostic investigations and being taken seriously) and the health service (timely, integrated care), shaped by prior healthcare experiences, patient symptom understanding, familiarity with medicine and uncertainty tolerance.

Participants emphasized the importance of receiving a diagnosis to gain understanding about the patient's health problem, consistent with previous research [2, 3, 5]. However, we found that while most participants *wanted* or *hoped* for a definitive diagnosis or explanation, many did not necessarily *expect* such outcomes. Prior ED visits may have influenced participants' expectations about the likelihood of receiving a definitive diagnosis. Many participants described factors contributing to uncertainty. Some explicitly distinguished between hoped‐for and expected outcomes regarding diagnosis, demonstrating they can coexist—patients may hope for a diagnosis, while resigned to possibly not receiving one. Drawing on previous healthcare experiences and medical knowledge, some participants demonstrated understanding of different types of uncertainty. They recognized aleatoric uncertainty—what is potentially knowable, e.g., through “further tests and research” (2301CA008), and epistemic uncertainty—the unknowable unknown; e.g., “sometimes they just don't know” (2301PA008) [47]. This novel finding extends previous research on expectations about diagnosis in EDs by applying healthcare expectations theory to begin to distinguish between hopes and expectations [21, 22], revealing greater complexity than previously documented.

Our findings show that patient and caregiver expectations about receiving a diagnosis or explanation existed on a certainty spectrum, with participants displaying a range of uncertainty awareness and tolerance. While most participants valued finding answers, many also anticipated ongoing uncertainty. Diagnostic investigations were commonly regarded as integral to understanding health problems. However, some participants recognized investigations may not resolve uncertainty, contrasting with a qualitative study reporting patients' belief “there is a test to diagnose the cause of every symptom.”^2(p540)^. Many participants expected doctors to rule out serious diagnoses to provide reassurance and/or working diagnoses rather than definitive answers, suggesting some regarded these as acceptable diagnostic endpoints in EDs, particularly when combined with guidance about next steps. This finding differs from prior research documenting that excluding serious diagnoses [44, 48, 49, 50], often via normal test results, may leave patients with ongoing uncertainty about the cause of symptoms [2, 11], causing dissatisfaction [51] and return visits [52, 53]. This discrepancy may reflect interview timing, as pre‐care interviews captured initial expectations rather than ongoing uncertainty post‐discharge.

Expectations about treatment and guidance about next steps similarly existed on a certainty spectrum. Some participants regarded diagnosis as a necessary precursor to these outcomes, echoing earlier qualitative findings [3] as to why patients seek diagnosis in EDs; however, others recognized treatment and guidance could be provided amid uncertainty. These findings suggest some participants viewed their ED visit as part of an ongoing diagnostic trajectory, demonstrating awareness of diagnosis as an iterative process unfolding over time, rather than the outcome of a single encounter—an expectation emergency doctors often regard as unrealistic [2, 17].

This concept of a certainty spectrum has important implications for how clinicians approach communication about expectations to build trust [54]. Although system pressures impact the time clinicians feel they can spend with patients, they should aim to spend ‘adequate time’ [55] on patient‐centered communication to understand individual expectations and uncertainty awareness, tailoring their approach accordingly. For patients and caregivers with limited awareness of uncertainty who expect a definitive diagnosis, acknowledging this expectation, understanding needs associated with seeking a diagnosis [3], and sharing information about the nature of diagnosis as a process and the diagnostic approach in EDs may help them understand likely diagnostic outcomes [56], including ongoing uncertainty [9]. For patients and caregivers with greater awareness of the diagnostic process and the impact of uncertainty on diagnostic outcomes, recognizing that hoped‐for and expected outcomes may co‐exist is crucial. While their expected diagnostic outcomes may align more closely with doctor's diagnostic goals [57, 58], they may be doing substantial work managing emotions related to hope and uncertainty—work that remains invisible to doctors. Recognizing that hoped‐for and expected diagnostic outcomes co‐exist creates opportunities for therapeutic listening, exploring and validating feelings about uncertainty to improve care experience and outcomes [59]. Frontloading communication about hopes, expectations and potential ongoing uncertainty, and revisiting this information at discharge [60] may better align expectations, and support patients and caregivers with ongoing uncertainty, than communicating uncertainty at discharge alone.

Participants' expectations about diagnostic investigations and timely, integrated care highlighted their priorities for achieving timely diagnostic outcomes in EDs. Previous research has documented the perception of EDs as a ‘one stop shop’ offering access to diagnostic investigations [25, 26, 27, 28] without the inconvenience and delays of multiple appointments across different sites [27, 28]. We found a similar perception among some participants, potentially shaped by prior ED visits, although others recognized that not all investigations could or would be performed during their ED visit—a sophisticated appreciation for the triage function of EDs. Such recognition aligns with the finding that some regarded ruling out serious conditions as an acceptable endpoint, when combined with clear guidance about next steps. Previous research also identified perceived urgency, anxiety, fear and reassurance‐seeking as drivers of ED (re)attendance [2, 25, 26, 27, 28, 52]. Our findings demonstrate patients and caregivers experiencing uncertainty make strategic decisions to present to ED that consider anticipated delays accessing community care and fragmented service delivery. While patients and clinicians may have different perceptions about symptom urgency [26, 61], timely, well‐founded reassurance (when warranted) is a genuine clinical need [28]. Normalizing help‐seeking through compassionate, respectful responses is therefore essential for patient safety, and aligns with public health messaging that encourages seeking medical advice for worrying symptoms [62].

For patients, being taken seriously is an important element of the diagnostic process in EDs [63, 64], especially when uncertainty persists. The prominence of this finding may reflect the high proportion of female patient participants, given gendered symptom invalidation, a form of testimonial epistemic injustice [65], is well‐documented [66, 67, 68]. Doctors less comfortable with uncertainty may respond in ways detrimental to patient‐centered diagnosis [69, 70, 71], such as dismissing patient symptoms [72], with potentially serious consequences for patient outcomes [73]. Bontempo et al.'s [73] recent meta‐synthesis of qualitative studies on difficult‐to‐diagnose illnesses demonstrated that patients experiencing symptom invalidation may subsequently under‐report information or avoid healthcare, and doctors may not adequately investigate symptoms—both potentially contributing to diagnostic delay. Our findings show patients, particularly those who have experienced invalidation, view thorough assessment as a marker of being taken seriously. In our study, some patients re‐presented to ED when they felt their concerns had not been addressed, echoing previous findings about repeat visits driven by unresolved uncertainty [2, 52]. Particularly amid uncertainty, thoroughness may provide value and reassurance to patients because it validates concerns. We are not advocating unjustified diagnostic testing to enhance patient perceptions of thoroughness. Rather, our findings underscore the importance of listening [59, 72, 74] and communicating about diagnostic reasoning [44], including which conditions were prioritized and ruled out (and why), and how these conclusions were reached. Careful listening and transparent reasoning that makes medical evidence accessible to patients may contribute to perceived thoroughness, helping patients know their concerns are taken seriously [63, 64] and facilitating trust [75, 76, 77, 78] when uncertainty persists.

### 4.1 Limitations

We focused on nonspecific symptoms among patients in selected triage categories. Patients with different symptoms, specific conditions or higher acuity may have different expectations. We collected data at two Australian public metropolitan teaching hospitals located in areas of socio‐economic advantage [79]. Patients presenting to hospitals in less advantaged areas, regional or rural settings, or private hospitals may have different expectations. Findings reflected features of Australia's public healthcare system and may differ in healthcare systems with different primary care models, referral pathways, access to diagnostic testing, or healthcare funding structures. Participants were predominantly female, English‐speaking and university‐educated, and all patients had previously presented to an ED, which may have influenced findings. The high proportion of female patients reflects response rates, higher female ED attendance [80], higher frequency of selected nonspecific symptoms in females [81, 82], and greater likelihood of receiving diagnoses classified as ‘symptoms, signs and abnormal findings’ (encompassing our selected nonspecific symptoms) [83]. Disparities in ED care based on gender, race and ethnicity are well‐documented [84, 85, 86, 87, 88], and potentially influence expectations, yet gender differences in reasons for seeking emergency care and expectations remain underexplored. Whether findings extend to broader populations, including first‐time ED users, requires further investigation. Purposive sampling for maximum variation could have addressed these limitations, but would have made data collection timeframes unfeasible as we followed patient journeys to admission or discharge [32]. Despite maintaining conversational privacy, conducting interviews in ED settings may have influenced how freely participants answered questions. Interview timing before initial consultation with an ED doctor may have colored tolerance for uncertainty overall.

We intended to compare patient and caregiver expectations but could not do so meaningfully given the small, diverse caregiver sample and the likely influence of the patient‐caregiver relationship on expectations. While sequential interview turn‐taking may have influenced individual responses, analysis of relational dynamics within patient‐caregiver pairs was beyond scope. Research on caregiver sub‐groups (spouses, adult children) and how the patient‐caregiver relationship shapes expectations is needed. Finally, LJC interviewed patients before their first consultation with an ED doctor to avoid influencing expectations. While methodologically important, unpredictable ED workflow created time constraints limiting the opportunity to explore some responses more deeply before a doctor arrived.

## 5 Conclusion

“Excellence in diagnosis means that the needs of the patient, for solace and relief, come first,” [89] placing patient‐centredness at the core of the diagnostic encounter. Understanding patient and caregiver needs is crucial to diagnostic excellence in emergency care [90], where patients, caregivers and doctors alike navigate diagnostic uncertainty. This study is the first to investigate expectations about diagnosis in EDs by interviewing participants during diagnostic uncertainty, situating expectations in their lived experience, before ED clinical influence. Findings revealed diverse expectations about diagnostic outcomes connected to awareness of the diagnostic process and its inherent uncertainty. Further patient‐centered research on uncertainty tolerance [70] and diagnostic communication deserves attention. Our findings highlight the importance of soliciting and responding to hopes and expectations in ways that address uncertainty inherent in the diagnostic process, given unmet expectations can undermine patient experience [13, 14, 15] and compromise safety through poor adherence to treatment and follow‐up [14, 16, 17]. Understanding patient and caregiver hopes and expectations enables doctors to communicate more effectively about diagnosis and the diagnostic process to enhance care and outcomes, even when uncertainty persists. Further research on patient preferences for communicating diagnostic uncertainty [56, 91], including in emergency care [92], is needed to guide clinical practice. Research comparing doctors' diagnostic goals with patient and caregiver expectations is needed to understand mismatches and guide interventions to improve communication about expectations in emergency care.

## Author Contributions

M.R.D. developed the study concept and design, extended by L.J.C. M.R.D. obtained funding. L.J.C. led the interviews with participants, using the interview guide developed by M.R.D. with input from J.M. and L.J.C. L.J.C. analyzed and interpreted the data with input from M.R.D., J.M., A.L., S.S., C.C. L.J.C. drafted the manuscript. All authors critically reviewed and approved the final manuscript.

## Funding

L.J.C.: ANU College of Arts and Social Sciences Kathleen Woodroofe PhD Scholarship in the Humanities/Social Sciences; Institute for Healthcare Improvement (IHI) Fellowship funded by the Gordon and Betty Moore Foundation and The John A. Hartford Foundation. M.R.D.: Australian Research Council, Discovery Early Career Researcher Award (DE220100785).

## Conflicts of Interest

The authors declare no conflicts of interest.

## Supporting information


**Appendix S1:** Interview guide.

**Appendix S2:** COREQ checklist.

**Appendix S3:** Individual participant characteristics.

## Data Availability

Research data are not shared due to privacy/ethics restrictions.
