# Supplementary material for: Beyond a Diagnosis: A Qualitative Study Exploring Patient and Caregiver Expectations About Emergency Department Visits Amid Uncertainty
Source: Acad Emerg Med. 2026 Jul 30;33(7):e70376. doi: 10.1111/acem.70376 (PMC13422665; doi:10.1111/acem.70376)
Supplement: Supplementary file 1 — Appendix S1: Interview guide. [file ACEM-33-0-s002.docx]

## Interview guide

1. Is this the first time that you’ve been to the Emergency Department (ED)?
2. Can you tell me what has brought you/the patient to the ED today (probe for main symptoms and/or main concern)
   1. If longstanding sign/symptom, 'what has changed to lead you/the patient to seek help in the ED today?'
3. What are your expectations from this visit? (probe What would be the best outcome for you/the patient?/ what would help you most/ What do you hope will come out of your ED visit today? )
4. Do you expect to be given an explanation for your/the patient’s symptoms (diagnosis) today?
   1. If yes, why?
   2. If no, why not?
   3. What do you think the explanation for your/the patient’s symptoms (diagnosis) might be?
5. Would you like your/the patient’s doctor to tell you about the reasons behind your/the patient’s diagnosis? (probe Would you like to know what the ED doctor is considering when trying to work out the problem?
6. How would you feel if you/the patient left without an explanation for your symptoms (a diagnosis) today?
7. Would you like your/the patient’s doctor to tell you about the different diagnoses they are considering?
   1. If yes, why?
   2. If no, why not?
8. Do you want your/the patient’s doctor(s) to tell you if they are uncertain about your/the patient’s diagnosis?
   1. If yes, why?
   2. If no, why not?
   3. additional probe questions Would you like the doctor to tell you the level of certainty they have when they give you a diagnosis? If yes, why? If no, why not?
   4. Would you be able to tell if your/the patient’s doctor(s) was/were uncertain about your/the patient’s diagnosis?
   5. If yes, how would you be able to tell?
   6. If no, what would the doctor need to do for you to think they were uncertain?
9. How would you feel if the doctor(s) told you/the patient they were uncertain about your diagnosis?

[further prompts]

- 1. Would it affect your expectations about coming to the ED today? If yes, how? If no, why not?
  2. Would it affect your thoughts about how competent the doctor(s) is/are? If yes, how? If no, why not?
  3. Would it affect your thoughts about the quality of care provided in the ED? If yes, how? If no, why not?

1. Would you question the doctor’s diagnosis, if you felt it wasn’t correct? (further prompt Or if you don’t understand how the diagnosis links to your symptoms?)
   1. If yes, how would you question it?
   2. How do you think they would react?
   3. If no, why not?
