# Supplementary material for: Beyond a Diagnosis: A Qualitative Study Exploring Patient and Caregiver Expectations About Emergency Department Visits Amid Uncertainty
Source: Acad Emerg Med. 2026 Jul 30;33(7):e70376. doi: 10.1111/acem.70376 (PMC13422665; doi:10.1111/acem.70376)
Supplement: Supplementary file 2 — Appendix S2: COREQ checklist. [file ACEM-33-0-s001.docx]

**Appendix 2. COREQ checklist**

| No. | Item | Description | Page/Response |
| --- | --- | --- | --- |
| Domain 1: Research team and reflexivity | | | |
| Personal Characteristics | | | |
| 1. | Interviewer/facilitator | Which author/s conducted the interview or focus group? | p.6, p.10 |
| 2. | Credentials | What were the researcher’s credentials? E.g. PhD, MD | LC - BA/BAs(Hons), GDipProfWrit  JM – GDipSciComm  AL – PhD  SS – Bmed  CC - MBBS  MRD – MA, PhD |
| 3. | Occupation | What was their occupation at the time of the study? | LC – PhD candidate  JM – Independent patient safety advocate  AL – Healthcare consumer  SS – ED Director  CC – ED Director  MD – Senior lecturer |
| 4. | Gender | Was the researcher male or female? | The research team included 5 females and 1 male |
| 5. | Experience and training | What experience or training did the researcher have? | p.8 |
| Relationship with participants | | | |
| 6. | Relationship established | Was a relationship established prior to study commencement? | p.6 |
| 7. | Participant knowledge of the interviewer | What did the participants know about the researcher? e.g. personal goals, reasons for doing the research | p.8 |
| 8. | Interviewer characteristics | What characteristics were reported about the interviewer/facilitator? e.g. Bias, assumptions, reasons and interests in the research topic | p.8 |
| Domain 2: Study Design | | | |
| Theoretical framework | | | |
| 9. | Methodological orientation and Theory | What methodological orientation was stated to underpin the study? *e.g. grounded theory, discourse analysis, ethnography, phenomenology, content analysis* | p.5 |
| Participant selection | | | |
| 10. | Sampling | How were participants selected? *e.g. purposive, convenience, consecutive, snowball* | p.6 |
| 11. | Method of approach | How were participants approached? *e.g. face-to-face, telephone, mail, email* | p.6 |
| 12. | Sample size | How many participants were in the study? | p.8-9  Table 2. |
| 13. | Non-participation | How many people refused to participate or dropped out? Reasons? | p.8 |
| Setting | | | |
| 14. | Setting of data collection | Where was the data collected? e.g. home, clinic, workplace. | p.6 |
| 15. | Presence of non-participants | Was anyone else present besides the participants and researchers? | p.6-7 |
| 16. | Description of sample | What are the important characteristics of the sample? *e.g. demographic data, date* | P8-9  Table 2. |
| Data collection | | | |
| 17. | Interview guide | Were questions, prompts, guides provided by the authors? Was it pilot tested? | p.7  Appendix 1 |
| 18. | Repeat interviews | Were repeat interviews carried out? If yes, how many? | No |
| 19. | Audio/visual recording | Did the research use audio or visual recording to collect the data? | p.7 |
| 20. | Field notes | Were field notes made during and/or after the interview or focus group? | p.5 |
| 21. | Duration | What was the duration of the interviews or focus group? | p.7 |
| 22. | Data saturation | Was data saturation discussed? | No, the concept of saturation is problematic. See Braun, V., & Clarke, V. (2021). To saturate or not to saturate? Questioning data saturation as a useful concept for thematic analysis and sample-size rationales. *Qualitative Research in Sport, Exercise and Health*, *13*(2), 201–216. https://doi.org/10.1080/2159676X.2019.1704846 |
| 23. | Transcripts returned | Were transcripts returned to participants for comment and/or correction? | p.7 |
| Domain 3: Analysis and Findings | | | |
| Data Analysis | | | |
| 24. | Number of data coders | How many data coders coded the data? | p.7-8 |
| 25. | Description of the coding tree | Did authors provide a description of the coding tree? | p.9.  Figure 1 |
| 26. | Derivation of themes | Were themes identified in advance or derived from the data? | p.7 |
| 27. | Software | What software, if applicable, was used to manage the data? | p.7 |
| 28. | Participant checking | Did participants provide feedback on the findings? | No, healthcare consumers in the research team did. p.7-8 |
| Reporting | | | |
| 29. | Quotations presented | Were participant quotations presented to illustrate the themes / findings? Was each quotation identified? *e.g. participant number* | p.10-16 |
| 30. | Data and findings consistent | Was there consistency between the data presented and the findings? | p.10-16 |
| 31. | Clarity of major themes | Were major themes clearly presented in the findings? | p.9-16 |
| 32. | Clarity of minor themes | Is there a description of diverse cases or discussion of minor themes? | p.13, p.15, p.16 |
