# Supplementary material for: Beyond a Diagnosis: A Qualitative Study Exploring Patient and Caregiver Expectations About Emergency Department Visits Amid Uncertainty
Source: Acad Emerg Med. 2026 Jul 30;33(7):e70376. doi: 10.1111/acem.70376 (PMC13422665; doi:10.1111/acem.70376)
Supplement: Supplementary file 3 — Appendix S3: Individual participant characteristics. [file ACEM-33-0-s003.docx]

**Appendix 3. Individual participant characteristics**

| Patient | Triage | Main presenting symptom | Caregiver | Relationship to patient | Medical knowledge | Gender | Age | Language | Ethnicity | Highest level of education completed |
| --- | --- | --- | --- | --- | --- | --- | --- | --- | --- | --- |
| 2301PA001 | 4 | Headache |  |  | Family member an ED doctor | Gender diverse | 26-35 | NES | Oceanian | University |
|  |  |  | **2301CA001** | Partner |  | Male | 18-25 | NES | Oceanian | University |
| 2301PA002 | 4 | Abdominal pain |  |  | Former radiographer | Female | 36-45 | NES | Oceanian | University |
| 2301PA003 | 4 | Dizziness |  |  |  | Female | 26-35 | NNES | Southern & Eastern Europe | University |
| 2301PA004 | 4 | Headache |  |  |  | Female | 26-35 | NES | Oceanian | High school |
| 2301PA005 | 4 | Abdominal pain |  |  |  | Female | 26-35 | NES | Oceanian | University |
|  |  |  | **2301CA005** | Spouse | Medical student | Female | 36-45 | NES | Oceanian | University |
| 2301PA007 | 4 | Headache |  |  |  | Female | 26-35 | NES | Oceanian | University |
| 2301PA008 | 3 | Abdominal pain |  |  | Studies in zoology | Female | 56-65 | NES | North American | University |
|  |  |  | **2301CA008** | Spouse |  | Male | 46-55 | NES | Oceanian | University |
| 2301PA009 | 4 | Abdominal pain |  |  |  | Male | 66-75 | NES | Oceanian | Vocational |
|  |  |  | **2301CA009** | Spouse | Retired nurse | Female | 66-75 | NES | Oceanian | University |
| 2301PA010 | 3 | Abdominal pain |  |  |  | Female | 36-45 | NES | Oceanian | High school |
|  |  |  | **2301CA010** | Paid carer |  | Female | 18-25 | NNES | Southern & Central Asia | University |
| 2301PA011 | 3 | Abdominal pain |  |  |  | Female | 56-65 | NES | Oceanian | Vocational |
|  |  |  | **2301CA011** | Friend |  | Female | 56-65 | NES | Oceanian | University |
| 2301PA012 | 3 | Chest pain |  |  |  | Female | 56-65 | NES | Sub-Saharan Africa | Vocational |
|  |  |  | **2301CA012** | Adult child | Some nursing training | Female | 26-35 | NES | Oceanian | Vocational |
| 2301PA013 | 3 | Abdominal pain |  |  |  | Female | 36-45 | NES | Oceanian | Vocational |
| 2301PAX02 | ^#^ | Abdominal pain |  |  |  | Male | >75 | NES | Oceanian | University |
|  |  |  | **2301CAX02A** | Adult child |  | Female | 46-55 | NES | Oceanian | University |
| 2301PAX03 | 3 | Chest pain |  |  | Nursing student | Female | 18-25 | NNES | Oceanian | High school |
| 2402PA001 | 3 | Headache |  |  |  | Female | 55-65 | NES | Oceanian | University |
| 2402PA002 | 4 | Abdominal pain |  |  |  | Female | 66-75 | NES | Oceanian | Some high school |
| 2402PA003 | 3 | Abdominal pain |  |  |  | Female | 46-54 | NES | Oceanian | University |
| 2402PA004 | 4 | Dizziness |  |  |  | Male | >75 | NES | Oceanian | Some high school |
|  |  |  | **2402CA004** | Friend |  | Female | 55-65 | NES | Oceanian | Vocational |
| 2402PA005 | 4 | Abdominal pain |  |  |  | Female | 66-75 | NES | Northwest European | University |
|  |  |  | **2402CA005** | Spouse |  | Male | 66-75 | NES | Northwest European | University |
| 2402PA006 | 4 | Abdominal pain |  |  |  | Female | 18-25 | NES | Indigenous Australian | University |
| 2402PA007 | 4 | Headache |  |  |  | Female | 36-45 | NNES | Northeast Asian | University |
| 2402PA008 | 3 | Dizziness |  |  |  | Female | 26-35 | NES | Sub-Saharan Africa | University |
|  |  |  | **2402CA008** | Spouse |  | Male | 26-35 | NES | Oceanian | University |
| 2402PA009 | 3 | Abdominal pain |  |  |  | Female | >75 | NES | Oceanian | Vocational |
| 2402PA010 | 3 | Dizziness |  |  | Retired nurse | Female | >75 | NES | Oceanian | Some high school |
|  |  |  | **2402CA010** | Friend |  | Female | 66-75 | NES | Oceanian | Some high school |

^#^Triage category unclear.

NES: Native English Speaker

NNES: Non-native English Speaker
